# [Retracted] MicroRNA‑326 inhibits melanoma progression by targeting KRAS and suppressing the AKT and ERK signalling pathways

**DOI:** 10.3892/or.2023.8488

**Published:** 2023-01-23

**Authors:** Kang Kang, Jing Zhang, Xiaoyun Zhang, Zhao Chen

Oncol Rep 39: 401–410, 2018; DOI: 10.3892/or.2017.6074

Following the publication of this paper, it was drawn to the Editors' attention by a concerned reader that various panels showing data from flow cytometric experiments in Figs. 2D, 5D and 6D, and the cell migration and invasion assay data shown in Fig. 2C and 5C, were strikingly similar to data appearing in different form in other articles by different authors. Moreover, the data appeared to be overlapping comparing between a pair of the panels in Figs. 2C and 5C, such that these may have been selected from the same original source, even though the data were intended to show the results from differently performed experiments. Owing to the fact that the contentious data in the above article were already under consideration for publication, or had already been published, elsewhere when it was submitted to *Oncology Reports*, the Editor has decided that this paper should be retracted from the Journal. After having been in contact with the authors, they agreed with the decision to retract the paper. The Editor apologizes to the readership for any inconvenience caused.

